# C/EBPβ is a MYB- and p300-cooperating pro-leukemogenic factor and promising drug target in acute myeloid leukemia

**DOI:** 10.1038/s41388-021-01800-x

**Published:** 2021-05-06

**Authors:** Maria V. Yusenko, Amke Trentmann, Debora A. Casolari, Luca Abdel Ghani, Mairin Lenz, Melanie Horn, Wolfgang Dörner, Stefan Klempnauer, Henning D. Mootz, Maria Francisca Arteaga, Jan-Henrik Mikesch, Richard J. D’Andrea, Thomas J. Gonda, Carsten Müller-Tidow, Thomas J. Schmidt, Karl-Heinz Klempnauer

**Affiliations:** 1 Institute for Biochemistry, Westfälische-Wilhelms-Universität, Münster, Germany; 2 Centre for Cancer Biology, SA Pathology and University of South Australia, Adelaide, SA Australia; 3 Institute for Pharmaceutical Biology and Phytochemistry, Westfälische-Wilhelms-Universität, Münster, Germany; 4 Department of Medicine V, Hematology, Oncology, Rheumatology, University Hospital Heidelberg, Heidelberg, Germany; 5 Department of Mathematics, Ruhr-Universität, Bochum, Germany; 6 Department of Medicine A, Hematology and Oncology, University Hospital, Westfälische-Wilhelms-Universität, Münster, Germany; 7 Cancer Research Institute, University of South Australia, Adelaide, SA Australia

**Keywords:** Acute myeloid leukaemia, Mechanisms of disease

## Abstract

Transcription factor MYB has recently emerged as a promising drug target for the treatment of acute myeloid leukemia (AML). Here, we have characterized a group of natural sesquiterpene lactones (STLs), previously shown to suppress MYB activity, for their potential to decrease AML cell proliferation. Unlike what was initially thought, these compounds inhibit MYB indirectly via its cooperation partner C/EBPβ. C/EBPβ-inhibitory STLs affect the expression of a large number of MYB-regulated genes, suggesting that the cooperation of MYB and C/EBPβ broadly shapes the transcriptional program of AML cells. We show that expression of *GFI1*, a direct MYB target gene, is controlled cooperatively by MYB, C/EBPβ, and co-activator p300, and is down-regulated by C/EBPβ-inhibitory STLs, exemplifying that they target the activity of composite MYB-C/EBPβ-p300 transcriptional modules. Ectopic expression of GFI1, a zinc-finger protein that is required for the maintenance of hematopoietic stem and progenitor cells, partially abrogated STL-induced myelomonocytic differentiation, implicating GFI1 as a relevant target of C/EBPβ-inhibitory STLs. Overall, our data identify C/EBPβ as a pro-leukemogenic factor in AML and suggest that targeting of C/EBPβ may have therapeutic potential against AML.

## Introduction

Transcription factor MYB is highly expressed in hematopoietic progenitor cells (HPCs) and performs key roles in their proliferation and differentiation by regulating the transcription of a plethora of target genes [1]. Recent progress in understanding its role in leukemia has made MYB a promising target for drug development [2]. Recurrent genomic alterations affecting MYB function have been identified in T-cell acute lymphoblastic leukemia and include amplifications or translocations of the *MYB* gene [3, 4] or result in the generation of de novo MYB-binding sites upstream of the *TAL1* or *LMO2* genes to stimulate their expression [5, 6]. In addition, Acute Myeloid Leukemia (AML) cells are “addicted” to high expression levels of MYB, making them more vulnerable to inhibition of MYB than normal HPCs [7–10]. This has further stimulated interest in MYB as a target for drug development as such a drug would allow the elimination of the leukemia cells while sparing normal hematopoiesis [2, 11]. Initial approaches based on small-molecule inhibitors of MYB have already yielded promising results, confirming that leukemia cells are more sensitive to targeting MYB than normal HPCs [12–19].

CCAAT-box/enhancer-binding protein beta (C/EBPβ) is a conserved leucine-zipper transcription factor that plays important roles in fundamental cellular processes including differentiation, proliferation, and growth arrest of specific cell types [20–22]. C/EBPβ is highly expressed in cells committed to the myelomonocytic hematopoietic lineage [23, 24] where it cooperates with MYB and the co-activator p300 to activate myeloid-specific gene expression [25–27]. Recent genome-wide binding studies have confirmed that MYB, C/EBPβ, and p300 co-localize at many promoters and enhancer sites in AML cells [28], suggesting that these proteins form a regulatory transcriptional module in myeloid cells.

Previously, we have characterized low molecular-weight compounds that inhibit MYB by disrupting its interaction with p300, providing the first evidence that MYB can be targeted by small-molecule inhibitors [13–15]. Subsequently, we have identified the natural sesquiterpene lactone (STL) 4,15-iso-atriplicolide tiglate (AT) and related STLs as novel inhibitors of MYB activity [29]. We have now characterized the inhibitory potential of these compounds in AML and show that they inhibit MYB indirectly by targeting its cooperation partner C/EBPβ. Our work highlights a novel role of C/EBPβ as a pro-leukemogenic factor and potential drug target for AML. Furthermore, we show that the growth factor independence 1 (*GFI1*) is a MYB-C/EBPβ-p300 target gene whose suppression by C/EBPβ-inhibitory STLs contributes to their ability to inhibit the proliferation of leukemia cells.

## Results

### Inhibition of C/EBPβ activity by 4,15-iso-atriplicolide-tiglate

**Fig. 1:**
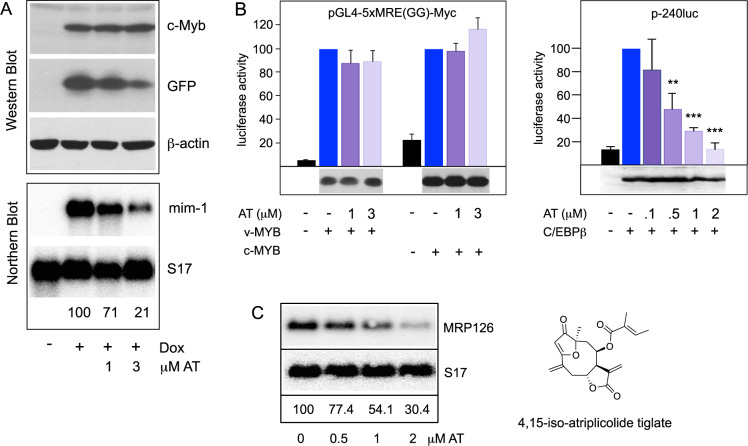
Inhibition of C/EBPβ activity by AT. **A** Inhibition of MYB-induced *MIM1* expression in HD11-C3-GFP1 cells by AT. Cells treated for 18 h with doxycycline and AT were analyzed by western blotting for MYB and GFP expression (upper panels) and by northern blotting for expression of the endogenous *MIM1* mRNA (lower panels). β-actin and S17 mRNA served as loading controls. The intensity of the mRNA bands was quantified with a phosphor-image analyzer. Numbers below the northern blots indicate the amount of *MIM1* mRNA relative to cells treated only with doxycycline. **B** Luciferase reporter experiments. QT6 fibroblasts were transfected with the MYB-dependent luciferase plasmid pGL4–5xMRE(GG)-Myc and expression vectors for v-MYB or chicken MYB (left) or with the C/EBPβ-inducible luciferase plasmid p-240luc and expression vector for chicken C/EBPβ (right). Cells were treated with AT and analyzed after 18 h. Co-transfection of the β-galactosidase expression vector pCMVβ was used to normalize luciferase activities. The bottom panels show the expression of MYB and C/EBPβ proteins. Asterisks indicate statistical significance (**p < 0.01, ***p < 0.001, Student’s *t* test). **C** QT6 fibroblasts were transfected with an expression vector for full-length C/EBPβ and treated for 18 h with the indicated concentrations of AT, as indicated at the bottom. RNA isolated from the cells was analyzed by northern blotting for the expression of *MRP126* mRNA. *S17* mRNA served as a loading control. The intensity of mRNA bands was determined as in (**A**). The numbers below indicate the expression levels of *MRP126* mRNA relative to the untreated control. The chemical structure of AT is shown on the right.

We have previously shown that several natural furanoheliangolide-type STLs possess MYB inhibitory activity in HD11-C3-GFP1 cells, a macrophage cell-line harboring a doxycycline-inducible MYB expression system and a stably integrated GFP reporter driven by the cis-elements of the MYB-inducible chicken *MIM1* gene [12, 29]. Figure 1A shows that the STL 4,15-iso-atriplicolide tiglate (AT) inhibits MYB-induced expression of the GFP-reporter as well as the endogenous *MIM1* gene in HD11-C3-GFP1 cells. Since *MIM1* expression requires the cooperation of MYB and C/EBPβ or C/EBPα, which are both expressed in HD11-C3-GFP1 cells [25, 30, 31], MYB-inhibitory compounds identified with this cell-system inhibit MYB itself or a cooperating C/EBP family member [32, 33]. We performed luciferase assays with either MYB- or C/EBP-dependent reporters to investigate if AT suppresses the activity of MYB or C/EBPβ. These experiments showed that AT inhibited C/EBPβ-activity but not MYB-activity (Fig. 1B). Additional reporter assays showed that the activity of C/EBPα was inhibited by AT only slightly (Supplementary Fig. 1). We also confirmed the inhibition of C/EBPβ at the endogenous *MRP126* gene, a physiological C/EBP target gene that is not expressed in fibroblasts but activated by exogenous C/EBPβ [34, 35] (Fig. 1C).

To corroborate the C/EBPβ-inhibitory potential of AT in a different biological context that is independent of MYB we examined its effect on the murine pre-adipocyte cell-line 3T3-L1, an established model of adipocyte differentiation. C/EBPβ controls a cascade of gene activation to induce an adipocyte phenotype that is visualized by the presence of cytoplasmic lipid vesicles and staining with the lipid dye Oil-Red-O [36]. AT strongly suppressed the differentiation of 3T3-L1 cells, further supporting the notion that AT disrupts C/EBPβ activity (Supplementary Fig. 2).

### Characterization of the C/EBPβ-inhibitory potential of AT-related STLs

As an initial step towards exploring structure-activity-relationships, we have investigated the inhibitory potential of the related compounds 4,15-iso-atriplicolide methacrylate (AMA), 4,15-iso-atriplicolide isobutyrate (AIB), budlein A (BUD), goyazensolide (GOY), 4,5-dihydro-atriplicolide-epoxyisobutyrate (DHA), and heliantuberolide-8-O-tiglate (HTT). All compounds inhibited the C/EBPβ-induced activation of the chromatin-embedded *MRP126* gene at EC50 concentrations around or slightly below 1 μM (Supplementary Fig. 3). This suggested that the α-methylene-γ-butyrolactone moiety as the only common group shared by these compounds is crucial for their inhibitory activity. As demonstrated before [33], α-methylene-γ-butyrolactone itself does not inhibit C/EBPβ, indicating that the presentation of this group as part of the furanoheliangolide skeleton increases its inhibitory potential towards C/EBPβ. Additional reactive groups, such as α,β- and α,β,γ,δ-unsaturated carbonyl groups or an epoxy-group found in some of the compounds may support or modify their inhibitory potential, making them interesting candidates for further studies.

The α-methylene-γ-butyrolactone group can react covalently with cysteine residues, raising the possibility that the furanoheliangolides inhibit C/EBPβ by covalent binding. Indeed, the inhibitory activity of AT was diminished by the thiol reagent dithiothreitol (Supplementary Fig. 4A), suggesting that the reaction of the compound with the thiol groups of DTT quenches its inhibitory activity. We employed mass spectrometry to obtain direct evidence for alkylation of C/EBPβ by AT. GFP-C/EBPβ isolated from cells treated with or without AT by binding to GFP-trap beads was therefore analyzed by LC-MS/MS. This identified a cysteine-containing peptide (amino acids 61–112) from the AT-treated sample whose mass showed the expected increase. Fragmentation of this peptide confirmed that AT was bound covalently to Cys-95 of C/EBPβ (Supplementary Fig. 5). However, a C/EBPβ mutant lacking Cys-95 remained sensitive to AT. We also showed that the cysteine-free C/EBPβ mutant CallA remained sensitive to AT (Supplementary Fig. 4B, C), indicating that the inhibition of C/EBPβ by AT was not caused by alkylation of cysteine residues of C/EBPβ.

Electrophoretic mobility shift assays with nuclear extracts from AT-treated cells showed no effect of AT on DNA-binding of C/EBPβ (Supplementary Fig. 6A). Furthermore, DNA-binding of C/EBPβ was also unaffected by up to 50 μM AT in the in vitro binding reaction (Supplementary Fig. 6B).

Previous studies had identified p300 as an important co-activator of C/EBPβ [30, 34, 35]. To see if AT affects the cooperation of C/EBPβ and p300 we examined its effect on the activation of the *MRP126* gene by C/EBPβ alone or combined with p300. Interestingly, p300 diminished the inhibitory effect of AT (Supplementary Fig. 7A) suggesting that AT disrupts the function of p300 as a co-activator of C/EBPβ. To investigate if p300 is targeted by AT we employed DARTS [37] (drug affinity responsive target stability), asking if incubation with AT in vitro affects the proteolytic stability of p300. DARTS assays can be used to identify potential drug targets by assessing their proteolytic stability. We employed p300 constructs containing or lacking the Taz2-domain that acts as a binding site for C/EBPβ. Supplementary Fig. 7B shows that incubation with AT strongly increased the sensitivity towards pronase digestion in the case of the p300 construct that contains the C/EBPβ binding region while it had no effect on the sensitivity of the construct lacking the Taz2 domain. Together, these data suggested that AT inhibits the activity C/EBPβ most likely via p300. However, the exact mechanism of inhibition and whether AT and related compounds act by covalent or non-covalent binding remains to be identified.

### Effects of 4,15-iso-atriplicolide derivatives on human myeloid leukemia cell lines and primary AML cells

**Fig. 2:**
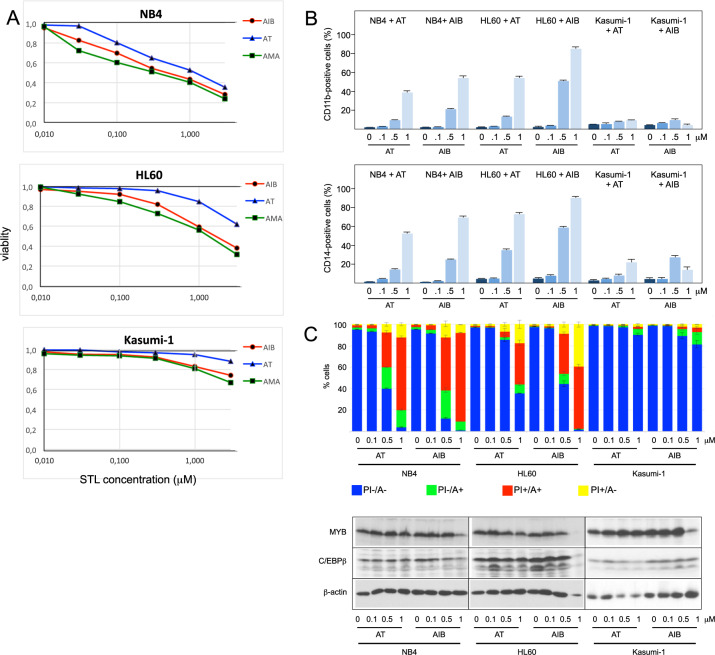
Effect of AT-related STLs on cell viability and induction of apoptosis and differentiation of myeloid leukemia cell lines. **A** NB4, HL60, and Kasumi-1 cells were treated with the indicated concentrations of AT, AMA, or AIB for 48 h. Cell viability was analyzed by a MTT assay. **B** NB4, HL60, and Kasumi-1 cells were treated for 2 days with the indicated concentrations of AT or AIB. The cells were then stained with antibodies against CD11b or CD14 and analyzed by flow cytometry. Bars indicate the percentage of positive cells with standard deviations. **C** Cells treated as in (**B**) were stained with annexin V-FITC (A) and propidium iodide (PI) and analyzed by flow cytometry. A-/PI-: living cells; A + /PI- and A + /PI + : early and late apoptotic cells; A-/PI + : necrotic cells. Bar charts indicate the percentage (with standard deviations) of living and apoptotic/necrotic cells of the whole cell population. The bottom shows western blot analyses of aliquots of the cells illustrating the expression of MYB, C/EBPβ, and β-actin.

Treatment of human myeloid leukemia cell lines NB4, HL60, and Kasumi-1 cells for 48 h with AT, AMA, or AIB showed a concentration-dependent decrease of viability in all cases (Fig. 2A). Analysis of differentiation markers CD11b and CD14 and staining with Annexin V and propidium iodide showed that AT and AIB induced expression of differentiation-associated genes and apoptosis in a concentration-dependent manner, albeit with cell line-specific efficiencies, without significant changes to MYB or C/EBPβ expression (Fig. 2B, C).

**Fig. 3:**
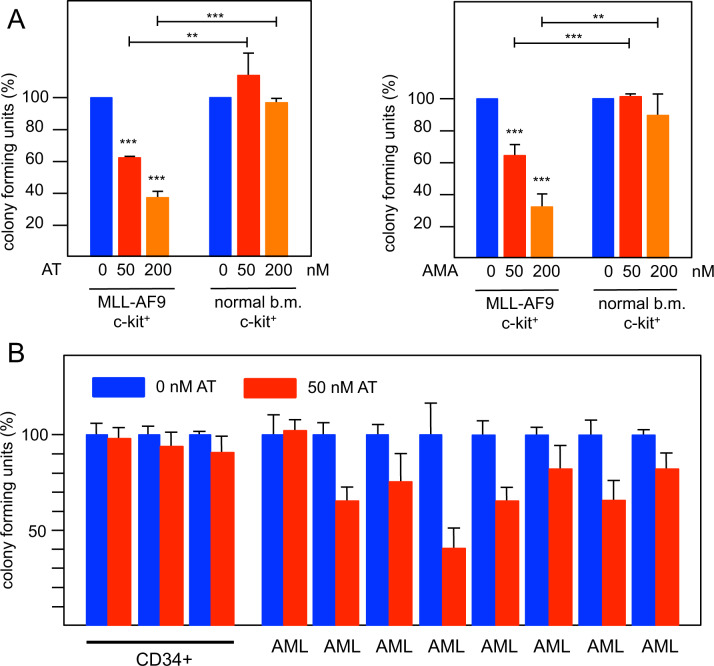
Effect of iso-atriplicolide derivatives on colony formation capacity of primary AML cells and normal hematopoietic progenitors. **A** Murine c-Kit positive cells from the bone marrow of healthy mice and murine MLL/AF9 AML cells were subjected to colony formation assays in the absence or presence of AT or AMA. Equal numbers of cells were plated with DMSO or with 50 or 200 nM AT or AMA. Columns show the relative colony number and standard deviation, normalized to the colony number of the DMSO control. Asterisks indicate statistical significance (*p < 0.05, ***p < 0.001, Student’s *t* test). **B** Colony formation assay of three independent pools of CD34 positive hematopoietic progenitor cells from healthy blood stem cell donors and leukemic blasts from 8 AML patients. Colony assays were performed with 50 nM AT as in A. All colony assays were performed in triplicates for each sample.

We used colony-forming assays to explore if iso-atriplicolide derivatives preferentially inhibit primary AML cells versus normal HPCs. Murine AML cells carrying the MLL-AF9 oncofusion were generated via retroviral transduction of murine c-Kit positive HPCs, injected into syngeneic mice, and recovered from the bone marrow of these mice after the development of AML [38]. Colony formation by murine AML cells was significantly reduced by AT or AMA at concentrations of 50 and 200 nM whereas normal c-Kit-positive HPCs isolated from the bone marrow of healthy mice were not affected significantly (Fig. 3A).

As a first step to explore the effect of AT on primary human AML cells, colony assays were performed with blasts from several AML patients and CD34 positive cells isolated from leukapheresis samples of healthy blood stem cell donors (Fig. 3B). We did not observe inhibition of colony formation in independent samples of cells from healthy donors at 50 nM AT. By contrast, in almost all cases colony formation from AML patient leukemic blasts was inhibited by 50 nM AT. Compared to the murine cells the response of AML patient cells was somewhat variable, presumably reflecting subtype specific responses due to the heterogeneity of the disease. Overall, these data suggest that primary murine and human AML cells on average are more sensitive to AT than normal HPCs. However, our data also demonstrate that the response of different AML cell-lines and cells from individual AML patients may be different, presumably because of distinct mutations giving rise to the disease.

### Treatment of AML cells ectopically expressing C/EBPβ or MYB shows that C/EBPβ is a relevant pro-leukemogenic MYB-cooperating factor

**Fig. 4:**
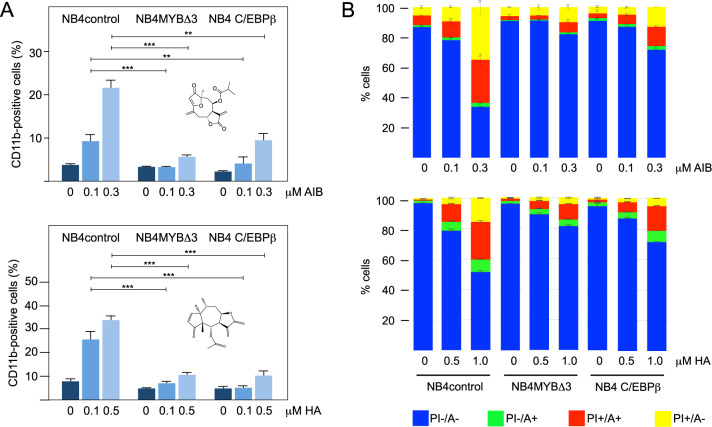
Ectopic expression of C/EBPβ or of MYB-Δ3 in NB4 cells counteracts AIB- and HA-induced differentiation and apoptosis. NB4 cells infected with a control lentivirus (NB4control), a MYBΔ3-encoding lentivirus (NB4MYBΔ3) or a C/EBPβ-encoding lentivirus (NB4C/EBPβ) were treated for 72 h with the indicated concentrations of AIB or HA. The cells were then stained with antibodies against CD11b (**A**) or with annexin V and propidium iodide (**B**) and analyzed by flow cytometry. Bars (**A**) indicate the percentage of CD11b positive cells (with standard deviations) of the viable cells. Asterisks indicate statistical significance (**p < 0.01, ***p < 0.001, Student’s *t* test). Bar charts (**B**) indicate the percentages (with standard deviations) of living (A-/PI-), early (A + /PI-) and late (A + /PI + ) apoptotic and necrotic (A-/PI + ) cells. The chemical structures of AIB and HA are shown in (**A**).

To further explore the mechanisms underlying the inhibitory effects of AT and its derivatives we investigated whether induction of differentiation and apoptosis was indeed mediated by inhibition of C/EBPβ. We employed NB4 cells, which showed a strong response to treatment with AT or AIB (Fig. 2), and infected them with a lentivirus encoding full length human C/EBPβ or with vector control. Treating the cells with AIB or helenalin acetate (HA), a C/EBPβ-inhibitory STL identified previously [32, 33], revealed that ectopic expression of C/EBPβ significantly diminished the induction of CD11b expression and apoptosis, consistent with the idea that these compounds exert their anti-proliferative effects via inhibition of C/EBPβ (Fig. 4A, B and Supplementary Fig. 8). Treatment of the cells with other furanoheliangolide derivatives including AT and GOY gave similar results (Supplementary Fig. 9). Thus, C/EBPβ appeared to be a relevant target of these compounds, whose inhibition is responsible for the induction of apoptosis and expression of differentiation-associated genes, suggesting a pro-proliferative role of C/EBPβ in AML cells.

We have previously shown that MYB, C/EBPβ, and co-activator p300 constitute a regulatory module of cooperating factors that drive myeloid-specific *MIM1* expression in chicken cells [25, 27, 34, 35]. Recent work has demonstrated co-localization of C/EBPβ, MYB, and p300 at multiple genomic sites in AML cells [28], implying that these factors also co-operate in AML and thus providing a conceptual framework for how C/EBPβ can contribute to the pro-leukemogenic function of MYB. We, therefore, wondered if C/EBPβ-inhibitory STLs indirectly also inhibit MYB as a consequence of its cooperation with C/EBPβ and whether increased MYB activity would dampen the inhibitory effects of the C/EBPβ-inhibitory STLs. To investigate this we used NB4 cells expressing a C-terminally truncated MYB, which acts as a stronger transcriptional activator than full-length MYB [39]. Interestingly, cells expressing MYB-Δ3 were rescued significantly from the induction of CD11b expression and apoptosis induced by AIB and HA (Fig. 4A, B) or related STLs (Supplementary Fig. 9), further supporting the notion that C/EBPβ is a relevant pro-leukemogenic cooperating factor of MYB in human AML cells.

### C/EBPβ-inhibitory STLs interfere with the expression of MYB target genes

**Fig. 5:**
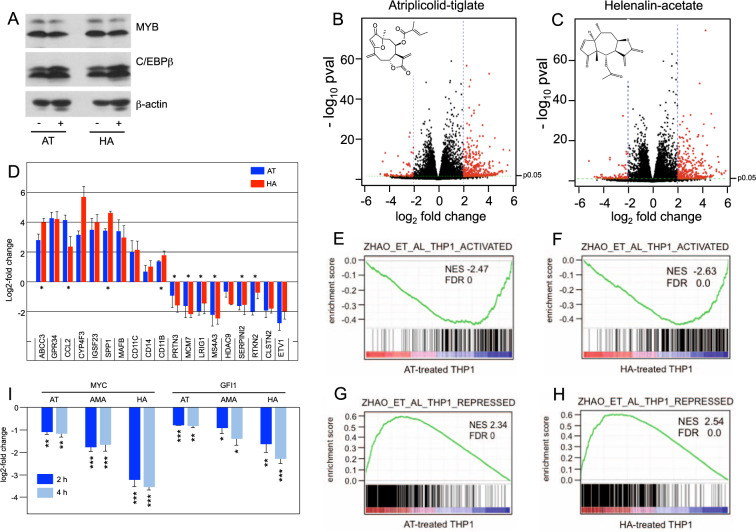
RNA profiling of AT- and HA-treated THP1 cells. **A** Western blot analysis of THP1 cells treated for 24 h without compound or with 1 μM AT or 0.5 μM HA. **B**, **C** Volcano plots showing gene expression changes (log2-fold change versus -log10 pval) of AT- and HA-treated THP1 cells. Genes up- or down-regulated four-fold or more (*p* < 0.05) are shown as red dots. **D** Real-time PCR of selected genes up- or down-regulated by AT or HA. Data are derived from three biological replicates. Bars indicate the log2-fold up- or down-regulation and standard deviation of the indicated genes. Known MYB-regulated genes are marked with asterisks. **E**–**H** GSEA of the AT- and HA-induced gene expression signature and genes repressed or activated by MYB in THP1 cells. **I** Decreased *MYC* and *GFI1* expression in THP1 cells after treatment for 2 and 4 h with AT (1 μM), AMA (1 μM), or HA (0.5 μM). Asterisks indicate statistical significance (*p < 0.05, **p < 0.01, ***p < 0.001, Student’s *t* test).

That C/EBPβ-inhibitory STLs disrupt MYB activity suggested that these compounds affect the expression of at least a fraction of MYB-regulated genes. To confirm this conjecture we treated THP1 cells for 24 h with 1 μM AT or 0.5 μM HA and compared their gene expression profiles to those of control cells. We used THP1 cells because previous work had already identified the gene expression changes induced by MYB silencing in THP1 cells [40], permitting direct comparison. The expression of MYB or C/EBPβ was not affected by treatment with the compounds (Fig. 5A). Considering only genes whose expression was determined with high confidence (padj < 0.05), RNA-Seq identified 615 and 275 genes, respectively, whose expression was up- or down-regulated two-fold or more by AT as well as 556 and 250 genes whose expression was up- or down-regulated at least two-fold by HA (Fig. 5B, C). Quantitative real-time PCR analyses of selected genes supported the validity of the RNA-Seq data (Fig. 5D and Supplementary Table 1). Importantly, there was a virtually complete overlap between the AT- and HA-regulated genes, as demonstrated by Gene Set Enrichment Analysis (GSEA) (Supplementary Fig. 10A, B). This was also evident from the real-time PCR analysis of selected HA-regulated genes (Fig. 5D). GSEA of the AT and HA gene expression signatures revealed that gene sets up-regulated by all-trans retinoic acid (ATRA) in HL60 cells or involved in myeloid development were enriched with genes up-regulated by AT and HA, confirming the induction of myeloid differentiation (Supplementary Fig. 10C-F). Furthermore, cytospin analyses of THP1 cells treated for 6 days with AT showed increased vacuolization and enlargement of the cells as early signs of differentiation (Supplementary Fig. 11). To assess the effect of AT and HA on MYB-regulated genes we employed gene sets of MYB-activated and MYB-repressed genes derived from knock-down of MYB in THP1 cells [40]. A large number of genes whose expression was stimulated by MYB were suppressed by AT and HA (Fig. 5E, F), and, vice versa, MYB-repressed genes were found enriched with AT- and HA-activated genes (Fig. 5G, H). This is also evident from the real-time PCR analyses in Fig. 5D, since several of the selected genes are known MYB-activated or -repressed genes (marked with asterisks). Thus, treatment of THP1 cells with either of the C/EBPβ-inhibitory compounds largely mimics the effect of MYB knock-down. As AT or HA do not inhibit MYB-activity directly (Fig. 1B and Ref. [32]) or decrease MYB expression (Fig. 5A) this suggested that these compounds exert their effects on MYB via inhibition of C/EBPβ, presumably as a result of the cooperation of MYB and C/EBPβ.

To exclude that the effects of both compounds were not caused indirectly by the differentiation of the cells we also examined the expression of *MYC* and *GFI1*, two direct MYB target genes [40, 41], after short-term treatment of THP1 cells with C/EBPβ-inhibitory STLs. Both genes were down-regulated already after 2 and 4 h of treatment, consistent with direct effects of the compounds on their expression (Fig. 5I). Taken together, our data showed that the inhibition of C/EBPβ by different STLs also inhibits MYB activity, consistent with the role of C/EBPβ as a crucial cooperation partner of MYB in AML cells.

### Forced expression of GFI1 prevents up-regulation of myelomonocytic differentiation markers CD11b and CD14 by C/EBPβ-inhibitory STLs

**Fig. 6:**
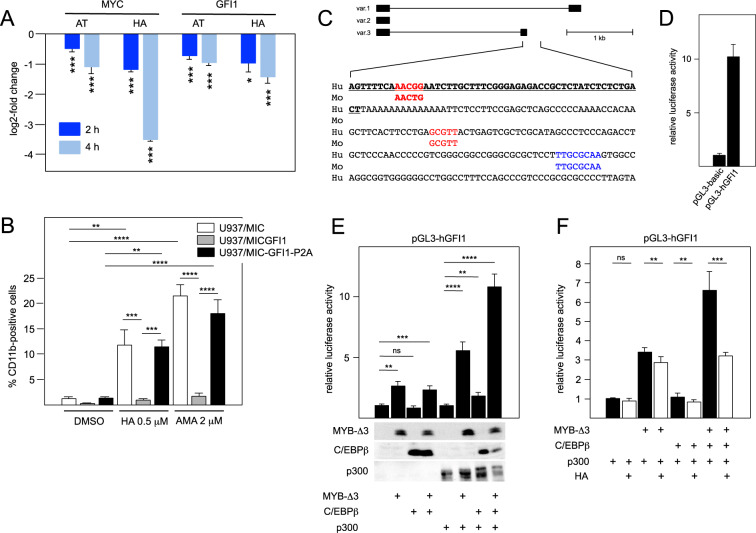
C/EBPβ-inhibitory STLs induce CD11b and CD14 expression in U937 cells by down-regulating *GFI1* expression. **A** Decreased *MYC* and *GFI1* expression in U937 cells after treatment for 2 and 4 h with AT (1 μM) or HA (0.5 μM). Statistical significance (*p < 0.05, ***p < 0.001, Student’s *t* test). **B** U937 cells expressing ectopic wild-type GFI1 (U937/MIC-GFI1), an inactive mutant of GFI1 (U937/MIC-GFI1-P2A) or no GFI1(U937/MIC) were analyzed by flow cytometry for CD11b expression. The bars indicate the percentage of CD11b positive cells after treatment for 4 days with DMSO, 0.5 μM HA or 2 μM AMA. Statistical significance was determined with one-way ANOVA with Tukey correction (*p < 0.05, **p < 0.01, ***p < 0.001, ****p < 0.0001). **C** Schematic illustration of *GFI1* transcript variants and nucleotide sequence of the *GFI1* variant 3 promoter region. Bold underlined letters correspond to the 5’ end of human *GFI1* variant 3 mRNA. Sequences in red and blue mark MYB and C/EBPβ binding sites, respectively. Conservation of these sequences in the mouse genome is indicated below. **D** Comparison of the luciferase activity of the cloned GFI promoter and the promoter-less luciferase vector. **E**, **F** Reporter assays of the human *GFI1* promoter in the presence of the indicated combinations of expression vectors for MYB-Δ3, C/EBPβ, and p300. In panel **E**, Western blotting (bottom) confirms the expression of the indicated proteins. Reporter assays in panel (**F**) were performed without or with 1 μM HA. Asterisks in panels (**E**) and (**F**) indicate statistical significance (*p < 0.05, **p < 0.01, ***p < 0.001, ****p < 0.0001, Student’s *t* test; *NS,* non-specific).

*GFI1* encodes a DNA-binding zinc finger protein that participates in the formation of repressive GFI1/CoREST chromatin complexes and is required for the maintenance of hematopoietic stem and progenitor cells [42–44]. MYB drives the expression of *GFI1* and thereby inhibits the differentiation of myelomonocytic cells [41]. We, therefore, wondered whether the down-regulation of *GFI1* expression by C/EBPβ-inhibitory STLs contributes to their ability to induce AML cell differentiation. To test this conjecture, we turned to U937 cells as a model of myelomonocytic differentiation to first confirm that these compounds were able to down-regulate *GFI1* (as well as *MYC*) expression in U937 cells after 2 or 4 h (Fig. 6A). We then employed previously described [41] U937 cells ectopically expressing wild-type GFI1, the inactive GFI1 mutant P2A or an empty vector control to examine whether forced GFI1 expression interferes with the induction of differentiation by AMA and HA. Both compounds increased the number of CD11b-positive U937 empty vector control cells. Importantly, this increase was largely suppressed by ectopic GFI1 while GFI1-P2A-expressing U937 cells showed similar numbers of CD11b-positive cells as the control (Fig. 6B and Supplementary Fig. 12A). CD14 levels also increased in the control cells upon treatment with both compounds and were suppressed by GFI1, but not by GFI1-P2A (Supplementary Fig. 12B, C). Because a significant percentage of the U937 cell lines were already CD14-positive without treatment with the compounds, these changes were most clearly demonstrated by analyzing the mean fluorescence intensity of the cell populations (Fig. 6B and Supplementary Fig. 12). Overall, these experiments indicated that GFI1 abrogates the induction of the CD11b and CD14 differentiation markers by the C/EBPβ-inhibitory compounds. Cytospin analyses of the cells treated for 4 days with HA or AMA showed morphological features of differentiation, irrespective of their GFI1 expression status (Supplementary Fig. 13). We, therefore, concluded that these compounds induce certain aspects of differentiation, such as increased CD11b and CD14 expression, by down-regulating *GFI1*, while other aspects were induced independently of GFI1.

Considering our hypothesis that C/EBPβ-inhibitory STLs inhibit MYB regulated genes by disturbing the cooperation of MYB, C/EBPβ, and p300, the down-regulation of *GFI1* by the C/EBPβ-inhibitory compounds implied that *GFI1* is a MYB-C/EBPβ-p300 target gene. ChIP experiments in mouse AML cells have demonstrated binding of C/EBPβ to sequences corresponding to the promoter region of one of the human *GFI1* transcript variants [28] (www.ncbi.nlm.nih.gov/genome/gdv/browser/geo/?id=GSE66122). The MYB binding site previously identified [41] is also located close to the promoter of this transcript variant (Fig. 6C). We have cloned the corresponding human promoter region and performed luciferase reporter assays (Fig. 6D, E). Co-transfection with expression vectors for MYB-Δ3, C/EBPβ, and co-activator p300 showed synergistic activation of the promoter when all three factors were expressed together. Interestingly, synergy of MYB-Δ3 and C/EBPβ was only observed in the presence of ectopically expressed p300. Together these data confirm that *GFI1* expression is controlled by the cooperation of MYB, C/EBPβ, and p300. Furthermore, reporter assays have confirmed the inhibitory activity of HA (Fig. 6F) or AMA (Supplementary. Fig. 14) when MYB, C/EBPβ, and p300 were co-expressed. Overall, our data establish *GFI1* as a relevant target of C/EBPβ-inhibitory STLs in myelomonocytic cells and support our model that these compounds target a pro-leukemogenic MYB-C/EBPβ-p300 transcriptional module.

## Discussion

AML is one of the most common types of leukemia in adults and children with poor outcomes, especially for the majority of elderly patients [45]. Improvement of existing therapies and development of novel therapeutic approaches for AML are therefore important goals. The identification of specific molecular dependencies of AML cells, including high MYB expression, has made MYB a promising drug target in AML [8, 10]. Importantly, the addiction of AML cells to higher levels of MYB compared to normal HPCs provides a rationale for a therapeutic approach based on MYB inhibition as it predicts leukemic cells to be more sensitive to inhibition of MYB than normal HPCs. This concept was validated by studies of a mouse model of AML and by initial work on small-molecule compounds targeting MYB. These studies confirmed that MYB-inhibitory compounds can significantly prolong leukemia latency in mouse models of AML [10, 12–19].

The characterization of several furanoheliangolide-type STLs that were initially found to inhibit MYB activity [29] confirmed and substantially extends our previous work on the STL helenalin acetate [32, 33] in demonstrating a key role of C/EBPβ as a pro-leukemogenic MYB-cooperating factor in AML cells. As shown here, these compounds do not affect MYB directly but rather inhibit the MYB-cooperating factor C/EBPβ, thereby causing up-regulation of differentiation-associated genes and apoptosis in human leukemia cell lines. Ectopic expression of C/EBPβ in the NB4 cell line reduces these anti-proliferative effects of AT and several related STLs, and confirms C/EBPβ as a relevant target of these compounds. Moreover, the observation that the effects elicited by AT derivatives or HA are dampened by the expression of an activated version of MYB strongly underlines the relevance of C/EBPβ as a pro-leukemogenic MYB-cooperating factor. Further support comes from the finding that treatment of AML cells with AT or HA affects the expression of a large number of MYB regulated genes. This is further exemplified by *MYC* and *GFI1*, two direct MYB target genes, whose expression was down-regulated by C/EBPβ-inhibitory STLs as early as after 2 h of treatment with the compounds. This indicates that the decreased expression of these genes is a direct effect and not caused indirectly by the differentiation of the cells. Overall, our data provide compelling evidence that C/EBPβ acts as a crucial pro-leukemic cooperation factor of MYB to maintain the survival and differentiation block of AML cells.

Previous work has established a role of C/EBPβ in promoting ATRA-induced APL cell differentiation [46], which appears inconsistent with the pro-leukemogenic function of C/EBPβ proposed here. However, ATRA-treatment of APL cells boosts C/EBPβ expression very strongly [46], raising the possibility that the high concentration of C/EBPβ in ATRA-treated cells leads to a transcriptional output that overrides its pro-leukemic function at lower concentrations of C/EBPβ. Concentration-dependent differential gene activation by a transcription factor is a common phenomenon, observed for example in many developmental processes. Possibly, ATRA-treatment also stimulates the expression of other factors that switch C/EBPβ from a pro-leukemogenic to a differentiation-inducing protein. This would also be consistent with our observation that overexpression of C/EBPβ alone is not sufficient to induce differentiation in NB4 cells (Fig. 4).

We presume that C/EBPβ acts in a MYB-C/EBPβ-p300 composite transcriptional module previously shown to control myeloid-specific gene expression in chicken cells [34]. This is supported by genome-wide binding studies showing that MYB, C/EBPβ, and p300 co-localize at many sites in the chromatin of AML cells [28]. We, therefore, hypothesize that the inhibition of C/EBPβ in the context of this module is responsible for differentiation and apoptosis triggered by C/EBPβ-inhibitory STLs. The analysis of the effect of these compounds on *GFI1* expression strongly supports this hypothesis and provides further mechanistic insight into the downstream effects elicited by C/EBPβ inhibition. *GFI1* plays an important role in maintaining the proliferation of hematopoietic stem and progenitor cells by forming repressive CoREST complexes that prevent the differentiation of immature myelomonocytic cells. Pharmacologic disruption of the interaction between GFI1 and LSD1, a major scaffolding and catalytic component of these complexes, induces differentiation in AML and has highlighted GFI1 and CoREST complexes as important therapeutic targets for the treatment of AML [47–50]. Consistent with our model, *GFI1* promoter studies reported here show that *GFI1* expression itself is controlled cooperatively by MYB, C/EBPβ, and p300. Importantly, we also identified *GFI1* as a relevant target of these compounds whose down-regulation contributes to their ability to induce myelomonocytic differentiation. Taken together, our data provide a consistent picture of C/EBPβ as a pro-leukemogenic factor and a relevant drug target in AML.

C/EBPβ-inhibitory STLs themselves may be interesting from a therapeutic perspective. As shown here, these compounds inhibit the proliferative potential of primary AML cells in a manner that parallels the higher dependency of these cells on MYB compared to normal HPCs. Thus, C/EBPβ-inhibitory STLs warrant further investigation of their potential as novel AML therapeutics and starting points for further drug development. Apart from AML, C/EBPβ has been implicated in the development of various malignancies, including multiple myeloma, ALK-positive anaplastic large cell lymphoma [51, 52], tumors of the colon, kidney, brain, stomach, prostate, and the ovaries [53–57] and its expression often correlates with increased malignancy and invasiveness of the tumor cells. Overall this makes C/EBPβ an interesting target for future drug development.

## Materials and methods

### Cells

HD11-C3-GFP1 reporter cells were described before [12]. Fat cell differentiation of the murine 3T3-L1 pre-adipocytic fibroblast line was induced as described previously [36]. HL60, NB4, Kasumi-1, U937, and THP1 are human myeloid leukemia lines. Cell lines were initially obtained from ATCC. U937 sub-lines U937/MIC, U937/MIC-GFI1, and U937/MIC-GFI1-P2A cells have been described [41]. All cell lines were free of mycoplasma contamination. Murine MLL-AF9 transformed AML cells were obtained as described before [14]. Murine hematopoietic c-Kit-positive progenitor cells were isolated from the femur of C57/BL6 wildtype mice and enriched on the basis of c-Kit expression by immunomagnetic (MACS Miltenyi Biotec) and c-Kit antibodies. Human acute myeloid leukemia (AML) blast cells were obtained from the bone marrow of patients diagnosed with AML at the Heidelberg University Hospital. Blasts were enriched (usually > 90%) by density centrifugation. CD34 + hematopoietic progenitor cells were isolated by magnetic cell sorting with anti-CD34 antibodies (MACS, Miltenyi Biotec) from leukapheresis samples of healthy donors undergoing harvest for allogeneic stem cell transplantation. All patients and donors provided written consent and the studies were approved by the local ethical board. Colony formation assays with murine and human AML cells and normal HPCs were performed as previously described [14, 58].

### Sesquiterpene lactones

4,15-iso-atriplicolide tiglate (AT), 4,15-iso-atriplicolide methacrylate (AMA), 4,15-iso-atriplicolide isobutyrate (AIB), and heliantuberolide-8-O-tiglate (HTT) was isolated from aerial parts of *Helianthus tuberosus* L. (Jerusalem Artichoke, Asteraceae) as described [59]. The origin of budlein A (BUD), goyazensolide (GOY), and 4,5-dihydro-atriplicolide-epoxyisobutyrate (DHA) have been described [29, 60]. The purity of all compounds was > 90% by HPLC and/or ^1^H-NMR. 10 mM stock solutions were prepared in DMSO.

### Viability, differentiation, and apoptosis assays

Cell viability was determined by MTT assays. Cells were incubated with compounds for 48 h, followed by addition of MTT solution (Millipore Corp., USA), incubation for 4 h, and measuring the absorbance at 495 nm with a microplate photometer (MPP 4008, Mikrotek). For differentiation and apoptosis assays cells were cultured for 2 days in RPMI 1640 medium containing the desired compounds and then analyzed for CD11b and CD14 expression using PE/Cy7-labeled anti-human CD11b (ICRF44, BioLegend) and FITC-labeled anti-human CD14 antibodies (63D3, BioLegend) and an FC 500 Cytometer (Beckman Coulter). Apoptotic and necrotic cells were determined by double-staining with FITC-annexin-V (BioLegend) and propidium iodide (PI). CXP software (Beckman Coulter) was used for subsequent analysis. Flow cytometry of GFI expressing U937 cells was performed by culturing the cells for 4 days with compounds at desired compound concentration or DMSO as control. Expression of CD11b and CD14 was measured using BB515 labeled anti-human CD11b (ICRF44, BD Biosciences) and BV421 labeled anti-human CD14 (M5E2, BD Biosciences) with the BD LSRFortessa Cell Analyzer. Subsequent analysis was performed using FCS Express 4 Flow Cytometry Research Edition (De Novo Software) and GraphPad Prism 8 (GraphPad Software). Cytospin slides were stained with May-Grunwald Giemsa.

Additional methods are described in Supplementary Materials.

## Supplementary information

Supplementary material
