# Supplementary material for: C/EBPβ is a MYB- and p300-cooperating pro-leukemogenic factor and promising drug target in acute myeloid leukemia
Source: Oncogene. 2021 May 6;40(29):4746–58. doi: 10.1038/s41388-021-01800-x (PMC8298201; doi:10.1038/s41388-021-01800-x)
Supplement: Supplementary file 1 — Supplementary material [file 41388_2021_1800_MOESM1_ESM.pdf]

Supplementary Material

C/EBPβ is a MYB-cooperating pro-leukemogenic factor and promising drug target in acute myeloid leukemia

Maria V. Yusenko[1], Amke Trentmann[1], Debora A. Casolari[2], Luca Abdel Ghani[1], Mairin Lenz[3], Melanie Horn[4], Wolfgang Dörner[1], Stefan Klempnauer[5], Henning D. Mootz[1], Maria Francisca Arteaga Paz[6], Jan-Henrik Mikesch[6], Richard J. D'Andrea[2], Thomas J. Gonda[7], Carsten Müller-Tidow[4], Thomas J. Schmidt[3] and Karl-Heinz Klempnauer[1]*

[1]Institute for Biochemistry, Westfälische-Wilhelms-Universität, Münster, Germany, [2]Centre for Cancer Biology, SA Pathology and University of South Australia, Adelaide, Australia, [3]Institute for Pharmaceutical Biology and Phytochemistry, Westfälische-Wilhelms-Universität, Münster, Germany, [4]Department of Medicine V, Hematology, Oncology, Rheumatology, University Hospital Heidelberg, Germany, [5]Department of Mathematics, Ruhr-Universität Bochum, Germany, [6]Department of Medicine A, Hematology and Oncology, University Hospital, Westfälische-Wilhelms-Universität, Münster, Germany, [7]Cancer Research Institute, University of South Australia, Adelaide, Australia.

Supplementary Methods

Transfections: QT6 cells were transfected by calcium-phosphate co-precipitation and reporter activities were analyzed as described previously[1]. The luciferase reporter pGL4-5xMRE(GG)-Myc contains 5 tandem copies of a Myb binding site fused to the human c-*myc* core promoter[2]. p-240luc contains the promoter of the chicken *MIM1* gene upstream of the luciferase gene[1]. Luciferase values were normalized against the β-galactosidase activity of the co-transfected plasmid pCMVβ (Clontech) to normalize the transfection efficiency. Reporter studies were performed in at least 3 independent experiments, with replicate transfections in each experiment. Expression vectors for v-MYB[3], chicken c-MYB[4], chicken C/EBPβ and C/EBPα[5], GFP-C/EBPβ and the cysteine-free C/EBPβ mutant CallA[6] have been described. The C95A mutant of chicken C/EBPβ was constructed by oligonucleotide-directed mutagenesis. Lentiviral vectors used are based on pLVX-dsRed (Clontech) which was modified by removing the RFP coding region (empty control vector) or replacing it with the coding regions for MYB-Δ3 (Uttarkar et al, 2016) or human C/EBPβ. Lentiviral particles were produced and used for infection as described previously[7].

Western and northern blotting: Cells were lysed in SDS-sample buffer to obtain total cell extract which was analyzed by SDS-polyacrylamide gel electrophoresis and western blotting with antibodies against MYB (clone 5E11)[8], chicken C/EBPβ[9], human C/EBPβ (sc-7962, Santa Cruz Biotechnology), rat C/EBPα[10], p300 (RW128, Thermo Fisher Scientific), β-actin (clone AC-15, Sigma-Aldrich) and GFP (clones 7.1 and 13.1, Roche Diagnostics). Isolation of polyadenylated RNA and northern blotting of *MIM1*, *MRP126* and *S17* mRNAs was performed as described[5,10]. mRNA bands were obtained by sequential hybridization of the same blots with different radiolabeled probes and were quantified with a phosphor image analyzer (Fujifilm BAS-1800II).

EMSA assays. Electrophoretic mobility shift assays were performed as described

previously[11] using radiolabeled complementary single-stranded oligonucleotides 5'-TGTAGCTGCAGATTGCGCAATCTGCATCTA-3' and 5'-GTAGATGCAGATTGCGCAATCTGCAGCTACA-3'. Nuclear extract was prepared from QT6 cells transfected with C/EBPβ expression vector treated for 18 hours without or with different concentrations of AT[11].

DARTS (drug affinity responsive target stability) assay[12]. Extracts of cells expressing the proteins of interest were prepared in ELB buffer (50mM Tris/HCl pH 7,5; 120mM NaCl; 1mM EDTA; 6mM EGTA; 0.5 % NP-40), supplemented with AT or DMSO and incubated on ice for 20 min. Aliquots were then incubated with serial dilutions of pronase for 15 min at room temperature and the reaction was stopped by adding a protease inhibitor cocktail containing PMSF, aprotinin, pepstatin A and leupeptin) and subsequent boiling in SDS-sample buffer. Input samples were kept on ice. The samples were then analyzed by SDS-PAGE and western blotting.

Quantitative real-time PCR: Total cellular RNA was isolated with TRIzol™ Reagent (Invitrogen) as recommended by the manufacturer. Total RNA (2 µg) was reverse-transcribed with the First Strand cDNA Synthesis Kit (K1612, ThermoScientific) using OligoT primers in 20 µL according to the manufacturer's instructions. Real-time RT-PCR reactions were carried out in 96-well plates using Power SYBR Green PCR Master Mix (Applied Biosystems). Reactions were performed using a StepOnePlus RT-PCR instrument (Applied Biosystems) and the following parameters: 95°C for 10 min, followed by 40 cycles of 95°C for 15 sec and 60°C for 60 sec. Each experiment included a no-template control. PCR reaction specificity was confirmed by melting curve analysis of the products. Primer sequences are given in supplementary table 1. Relative gene expression was calculated by the $\Delta\Delta C_T$ method[13]: First, ΔC_T values were calculated by subtracting the C_T-values obtained for individual mRNAs from those obtained for β-actin mRNA. Then, $\Delta\Delta C_T$ values were calculated by subtracting the ΔC_T values of AT-treated cells from those of untreated cells. All experiments were conducted with at least three biological replicates.

Analysis of C/EBPβ by LC-MS/MS. QT6 cells were transfected with an expression vector for GFP-C/EBPβ and treated for 18 hours in the presence or absence of 1 μM AT. After lysis in ELB buffer (50mM Tris/HCl pH 7,5; 120mM NaCl; 1mM EDTA; 6 mM EGTA; 0.5 % NP-40) GFP-C/EBPβ was recovered from the cell extract using GFP-trap beads (Chromotek). The beads were washed three times with ELB and twice with NH_4HCO_3-solution (100 mM). Supernatant was discarded and the beads with bound proteins were incubated in 20 µl DTT (1 mM in 100 mM NH_4HCO_3) for 30 min at 56 °C followed by an alkylation step (30 minutes at RT) by adding 10 µl of 2-iodoactamide solution (10 mM in 100 mM NH_4HCO_3). Beads were washed twice with ddH_2O and resuspended in 40 µL digestion solution (2 ng Trypsin, 50 mM NH_4HCO_3, 0.75% (v/v) ProteaseMax). The samples were incubated for 120 min at 37 °C and the supernatant transferred. LS/MS^2 analysis of 40 µL peptide solution was performed using an UltiMate™ 3000 RS LC nano system (Thermo Fisher Scientific GmbH, Dreieich, Germany) connected to a maXis II UHR-qTOF mass spectrometer with a nano-ESI source (CaptiveSpray with nanoBooster, Bruker Daltonik GmbH, Bremen, Germany). The sample was acidified with formic acid to a final concentration of 0.1%, loaded on a C18 trapping column (Acclaim PepMap100, 5 µm, 100 Å, ID 300 µm x L 5 mm, Thermo Fisher Scientific GmbH, Dreieich, Germany) at a flow rate of 20 µL/min in 2% eluent B (eluent A: 0.1% formic acid in water; eluent B: 0.1% formic acid in acetonitrile). After 5 minutes of washing at 2% B a two step gradient (120 minutes from 2% to 50% B; 10 min from 50% to 95%, flow rate 300 nL/min) was applied for the separation on a C18 nano column (Acclaim™ PepMap™ 100 C18, 2 µm, 100 Å, ID 300 µm x L 150 mm, Thermo Fisher Scientific GmbH, Dreieich, Germany). MS settings: capillary voltage 1200 V, mass range: m/z 150-2200. MS survey scans were performed with a cycle time of 3 s. After each survey scan, the 10 to 20 most abundant precursor ions with $z > 1$ were selected for fragmentation using collision-induced dissociation. MS/MS summation time was adjusted depending on the precursor intensity, the precursor isolation window and the collision energy were depending on the precursor m/z and charge. DataAnalysis 4.4

(Bruker Daltonik GmbH, Bremen, Germany) was used for chromatogram processing and fragment spectra isolation. The resulting data were analyzed using ProteinScape 4 (Bruker Daltonik GmbH, Germany) as a front-end for searches against in-house databases on a Mascot server (Mascot 2.5, Matrix Science Ltd., London, UK) containing the sequence of chicken C/EBPβ. Cysteine carbamidomethylation and cysteine modification by AT were chosen as variable modifications for database searching.

RNA-Seq: Total cellular RNA was isolated with TRIzol™ Reagent (Invitrogen). RNA quality was controlled with a Bioanalyzer (Agilent), followed by poly(A) enrichment using the NEBNext Poly(A) enrichment kit. RNA-Seq libraries were constructed using the NEBNext Ultra Directional RNA II Library Prep kit. The quality of the library was checked using the TapeStation System (Agilent) and quantified with the NEBNext Library Quant Kit. Barcoded RNA-Seq libraries were sequenced with the Illunmina NextSeq 500 System and the NextSeq v2 sequencing reagent kit in the single-read mode with a read-length of 75 bp. The sequences were demultiplexed (bcl2fastq2) to fastq data and quality controlled (https://www.bioinformatics.babraham.ac.uk/projects/fastqc/). Trimmomatic (version 0.38) was used for adapter trimming and read filtering[14]. The resulting reads were aligned to the Ensembl GRCh38 reference genome using Hisat2 (version 2.1.0)[15] and sorted using SAMtools (version 1.9)[16]. The sorted and aligned reads were counted into genes using HTSeq (version 0.11.1)[17]. Testing for differential expression was performed using the DEseq2 r-package (version 1.22.2)[18]. GSEA was performed according to (www.broadinstitute.org/gsea/index.jsp) using gene-sets from the MSigDB[19] or DsigDB[20] database.

Statistical analysis: All experiments subjected to statistical analysis were performed at least three times with independent replicates in each experiment. Data were shown as mean ± standard deviation, which reflects the variation within each group. Statistical differences between groups were calculated by the two-tailed Student's *t*-test or by one-

way ANOVA with Tukey correction. Values of $P < 0.05$ were considered as statistically significant.

Data sharing statement: RNA-Seq data are available at GEO under accession number GSE128570.

Supplementary References

1. Chayka O, Kintscher J, Braas D, Klempnauer K-H. v-Myb mediates cooperation of a cell-specific enhancer with the *mim*-1 promoter. *Mol. Cell. Biol.* 2005;25(1):499-511.

2. Molvaersmyr AK, Saether T, Gilfillan S, et al. A SUMO-regulated activation function controls synergy of c-Myb through a repressor-activator switch leading to differential p300 recruitment. *Nucleic Acids Res.* 2010;38(15):4970-4984.

3. Ivanova O, Braas D, Klempnauer K-H. Oncogenic point mutations in the Myb DNA-binding domain alter the DNA-binding properties of Myb at a physiological target gene. *Nucleic Acids Res.* 2007;35(21):7237-7247.

4. Singh P, Wedeken L, Waters LC, Carr MD, Klempnauer KH. Pdcd4 directly binds the coding region of c-myb mRNA and suppresses its translation. *Oncogene*.2011;30(49):4864-4873.

5. Steinmann S, Coulibaly A, Ohnheiser J, Jakobs A, Klempnauer KH. Interaction and cooperation of the CCAAT-box enhancer-binding protein β (C/EBPβ) with the homeodomain-interacting protein kinase 2 (Hipk2). *J. Biol. Chem*. 2013;288(31):22257-22269.

6. Jakobs A, Steinmann S, Henrich SM, Schmidt TJ, Klempnauer K-H. Helenalin acetate, a natural sesquiterpene lactone with anti-inflammatory and anti-cancer activity, disrupts the cooperation of CCAAT-box/enhancer-binding protein beta (C/EBPβ) and co-activator p300. *J. Biol. Chem*. 2016;291(50):26098-26108.

7. Uttarkar S, Dasse E, Coulibaly A, et al. Targeting acute myeloid leukemia with a small molecule inhibitor of the Myb/p300 interaction. *Blood.* 2016;127(9):1173-8211.

8. Sleeman JP. Xenopus A-myb is expressed during early spermatogenesis. *Oncogene*. 1993;8(7):1931-1941.

9. Mink S, Kerber U, Klempnauer K-H. Interaction of C/EBPbeta and v-Myb is required for synergistic activation of the mim-1 gene. *Mol. Cell. Biol.* 1996;16(4):1316-1325.

10. Burk O, Mink S, Ringwald M, Klempnauer K-H. Synergistic activation of the chicken *mim-1* gene by v-*myb* and C/EBP transcription factors. *EMBO J.* 1993;12(5):2027-2038.

11. Plachetka A, Chayka O, Wilczek C, Melnik S, Bonifer C, Klempnauer K-H. C/EBPbeta induces chromatin opening at a cell-type-specifc enhancer. *Mol. Cell. Biol.* 2008;28(6):2102-2112.

12. Lomenick B, Jung G, Wohlschlegel JA, Huang J. Target identification using drug affinity responsive target stability (DARTS). *Curr. Protoc. Chem. Biol*. 2011;3:163-180.

13. Litvak KJ, Schmittgen TD. Analysis of Relative Gene Expression Data Using Real-Time Quantitative PCR and the $2^{-\Delta\Delta CT}$ Method. *Methods* 2001; 25:402-408.

14. Bolger AM, Lohse M, Usadel B. Trimmomatic: a flexible trimmer for Illumina sequence data. *Bioinformatics*. 2014;30(15):2114-2120.

15. Kim D, Langmead B, Salzberg SL. HISAT: a fast spliced aligner with low memory requirements. *Nat. Methods*. 2015;12(4):357-360.

16. Li H. A statistical framework for SNP calling, mutation discovery, association mapping and population genetical parameter estimation from sequencing data. *Bioinformatics*. 2011;27(21):2987-2993.

17. Anders S, Pyl PT, Huber W. HTSeq--a Python framework to work with high-throughput sequencing data. *Bioinformatics*. 2015;31(2):166-169.

18. Love MI, Huber W, Anders S. Moderated estimation of fold change and dispersion for RNA-seq data with DESeq2. *Genome Biol*. 2014;15(12):550.

19. Subramanian A, Tamayo P, Mootha VK, et al. Gene set enrichment analysis: A knowledge-based approach for interpreting genome wide expression profiles. *Proc. Natl. Acad. Sci. USA*. 2005;102(43):15545-15550.

20. Yoo M, Shin J, Kim J, et al. DSigDB: drug signatures database for gene set analysis. *Bioinformatics*, 2015;31(18):3069–3071.

Supplementary Figures

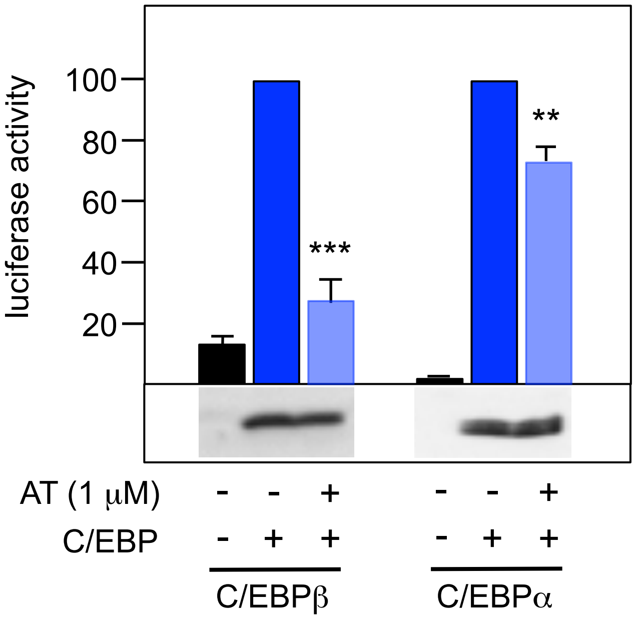

Supplementary Fig.1. **Inhibition of C/EBPβ and C/EBPα by AT**. QT6 cells were transfected with expression vector for full-length chicken C/EBPβ or chicken C/EBPα. C/EBP levels are shown at the bottom. Asterisks indicate statistical significance (**p<0.01, ***p<0.001, Student's t-test).

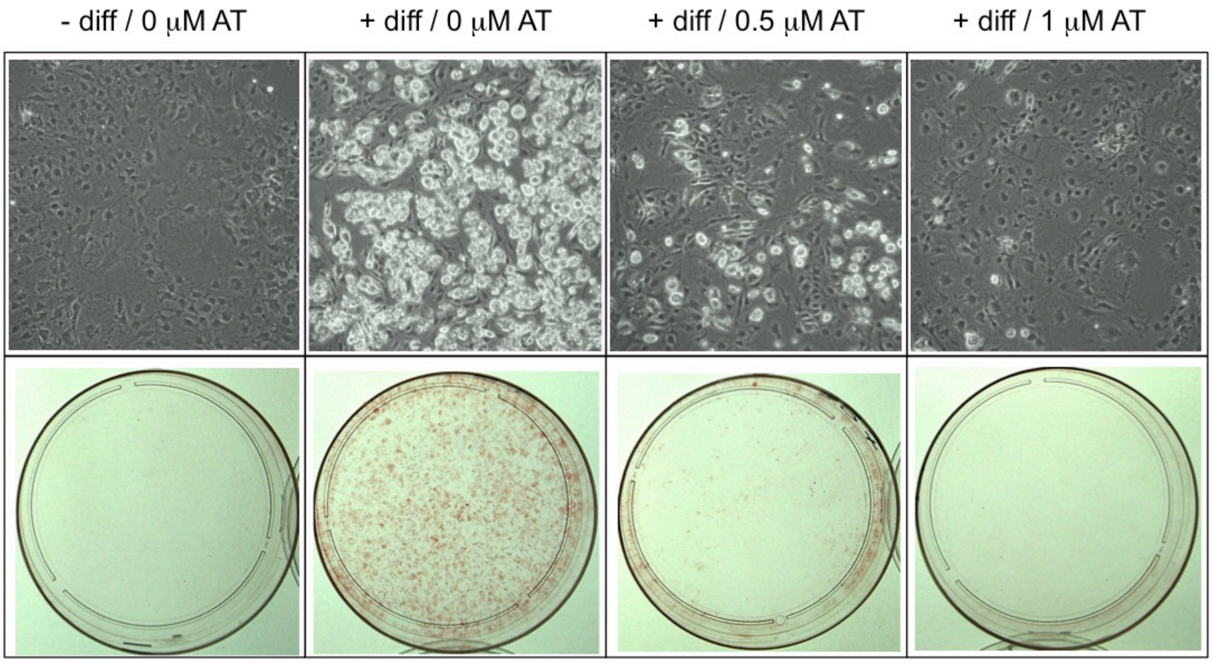

Supplementary Fig.2. **iso-atriplicolide tiglate inhibits adipocyte differentiation**. Upper part: Photomicrographs of 3T3-L1 cells without induction of differentiation or induced to undergo adipocyte differentiation for 7 days, either in the absence or the presence of the indicated concentrations of AT. Lower part: Oil-Red-O staining of tissue culture dishes treated as indicated at the top.

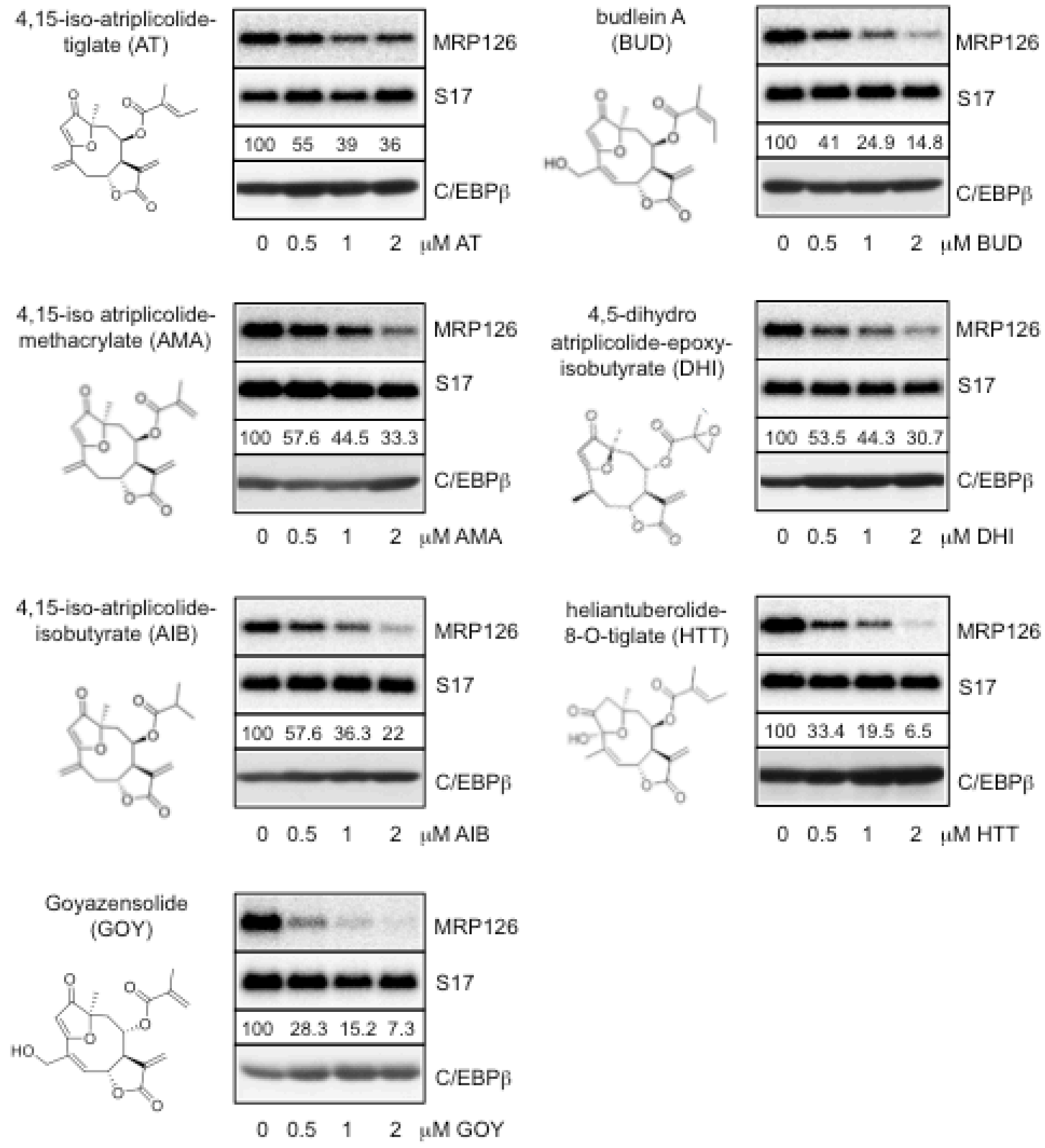

Supplementary Fig.3. **Characterization of the inhibitory potential of furanoheliangolide STLs**. **A.** QT6 cells were transfected with expression vector for chicken C/EBPβ and treated for 18 hours with different concentrations of the indicated compounds. RNA isolated from the cells was analyzed by northern blotting for the expression of *MRP126* mRNA (top panels). *S17* mRNA served as loading control (middle panels). The intensity of the bands was quantified with a phosphor-image analyzer and the numbers below the top two panels indicate the expression levels of *MRP126* mRNA normalized against the *S17* mRNA levels and relative to the untreated control. The signals for *MRP126* and *S17* mRNAs were obtained by sequential hybridization of the same blots with specific radiolabeled probes. The bottom panels show C/EBPβ expression.

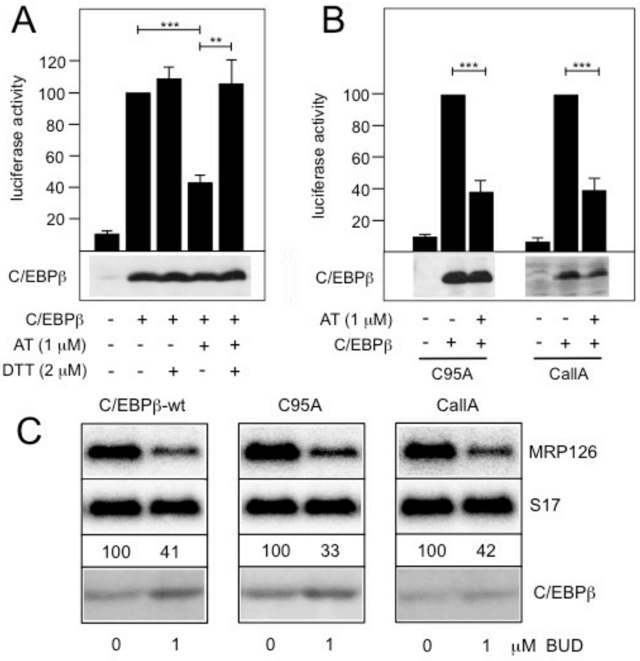

Supplementary Fig.4. **Alkylation of C/EBPβ by AT**. **A.** QT6 cells transfected with the reporter plasmid p-240Luc and expression vector for C/EBPβ were cultivated for 18 hours with different combinations of AT and DTT, as indicated at the bottom. Reporter activities were analyzed as in Fig.1. Columns show the normalized luciferase activities with standard deviations. Asterisks indicate statistical significance (**p<0.01, ***p<0.001, Student's t-test). The expression of C/EBPβ is shown at the bottom. **B.** QT6 cells were transfected as in A except that expression vectors for C/EBPβ-C95A and C/EBPβ-CallA were used and DTT was omitted. Luciferase activities were analyzed as in A. **C.** QT6 cells were transfected with the indicated C/EBPβ expression vectors and treated for 18 hours without or with BUD. RNA was then analyzed by northern blotting for the expression of *MRP126* mRNA. *S17* mRNA served as loading control. The intensity of the bands was quantified with a phosphor-image analyzer and the numbers below the top two panels indicate the expression levels of *MRP126* mRNA normalized against the amount of *S17* mRNA and relative to the untreated control. The C/EBPβ expression levels are shown at the bottom.

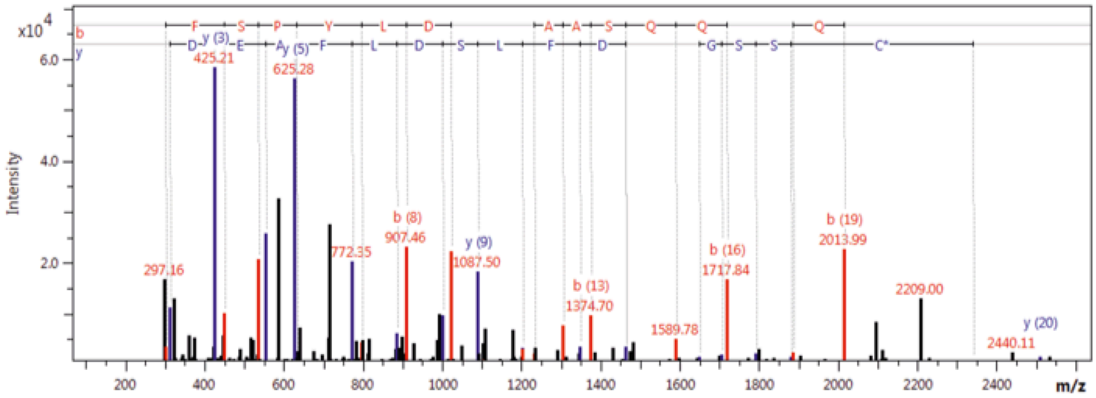

AS 61-112: AIDFSPYLDPLAASQQPAQPPPPAAAAGGNFEPA**C**SSGGQDFLSDLFAEDYK

m/z of 5fold charged peptide: 1425.1589 (observed); 1425.16063 (calculated)

Supplementary Fig.5**. iso-atriplicolide tiglate alkylates Cys-95 of chicken C/EBPβ**. Collision-induced fragmentation pattern of a peptide from chicken C/EBPβ showing covalent binding of AT to Cys-95. The precursor peptide corresponds to amino acids 61-112 of chicken C/EBPβ. The amino acid sequence of the presursor peptide and the observed and calculated m/z values for the AT-modified peptide are indicated at the bottom.

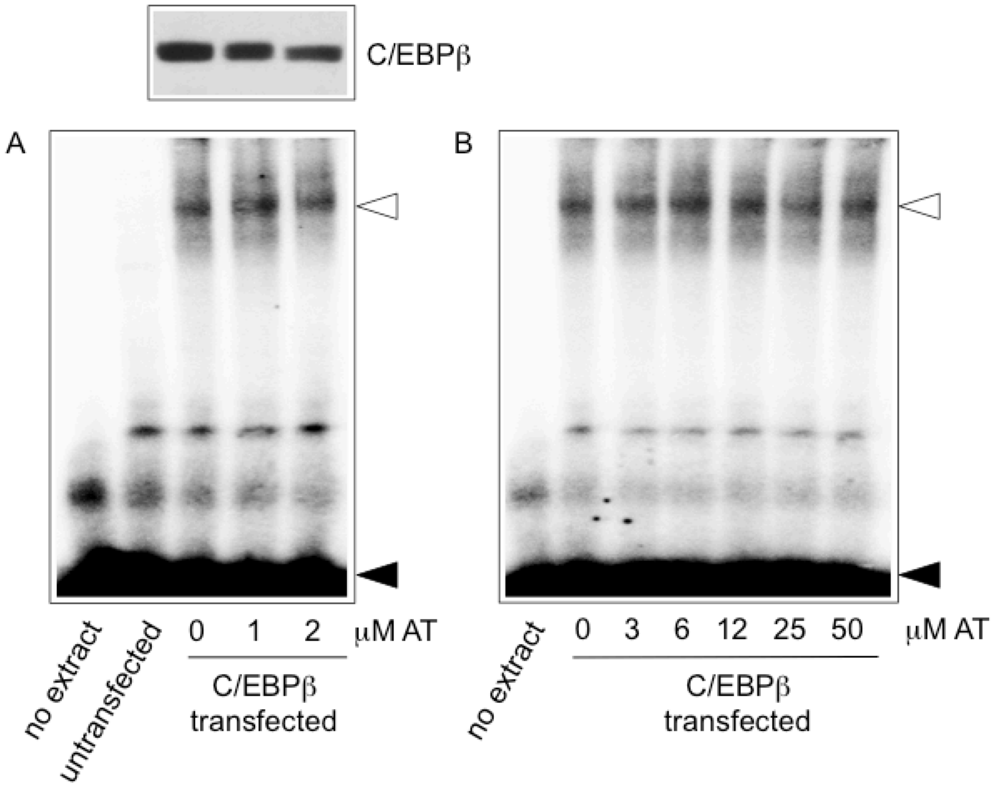

Supplementary Fig.6. **AT does not inhibit DNA-binding by C/EBPβ. A.** A radiolabeled double-stranded C/EBP consensus binding site oligonucleotide was incubated without or with nuclear extract from cells transfected with C/EBPβ expression vector and treated with AT or from untransfected cells, as indicated. Protein-DNA complexes were displayed by native polyacrylamide gel electrophoresis. White and black arrowheads mark protein-DNA-complexes and free oligonucleotide, respectively. The top panel shows a western blot analysis of aliquots of the nuclear extracts stained with antibodies against C/EBPβ. **B.** Nuclear extract of C/EBPβ-transfected but AT untreated cells was incubated with the oligonucleotide used in A and the indicated concentrations of AT in the binding reaction. Protein-DNA-complexes were analyzed as in A.

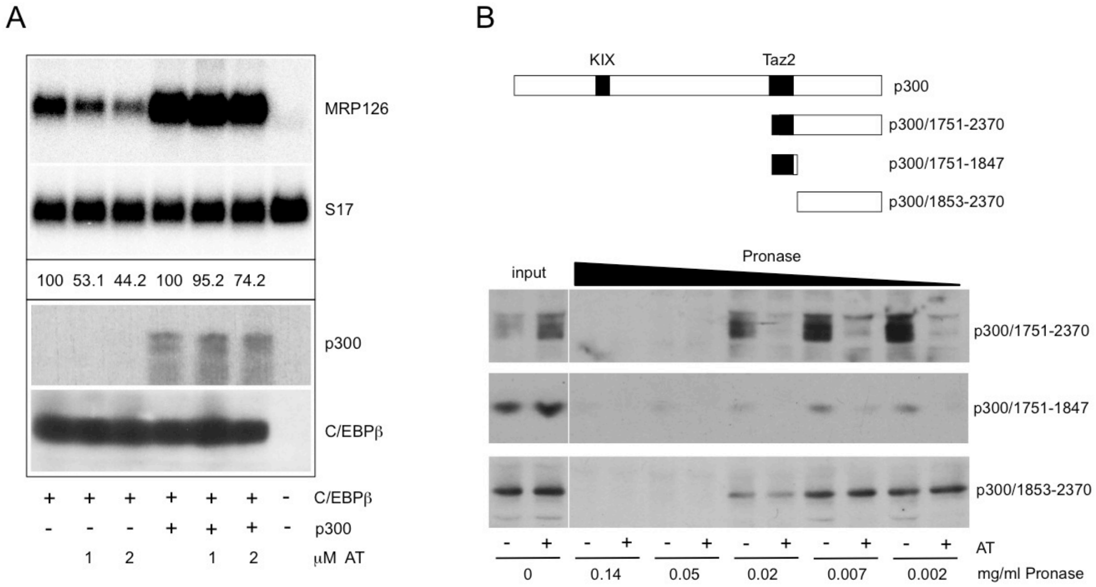

Supplementary Fig.7. **AT disrupts the function of p300 as a coactivator of C/EBPβ. A.** QT6 cells transfected with expression vectors for C/EBPβ and p300 were treated with the indicated concentrations of AT and harvested after 18 hours. RNA was isolated and analyzed by northern blotting for the expression of *MRP126* mRNA. *S17* mRNA served as loading control. The intensity of the bands was quantified with a phosphor-image analyzer and the numbers below the top two panels indicate the expression levels of *MRP126* mRNA normalized against the amount of *S17* mRNA and calculated relative the untreated controls. Aliquots of the transfected cells were analyzed by western blotting for the expression of C/EBPβ and p300 (lower panels). **B.** Cell extracts expressing the p300 constructs shown schematically at the top were subjected to DARTS assays. Aliquots of the cell extracts supplemented with 100 mM AT (+) or the equivalent amount of solvent (-) were incubated with serially diluted amounts of pronase for 15 min at room temperature. Input samples were kept on ice. Proteins were analyzed by SDS-PAGE and western blotting with bodies against p300 (top and bottom panels) or against the HA-tag (middle panel). Taz2 refers to the C/EBP binding site of p300.

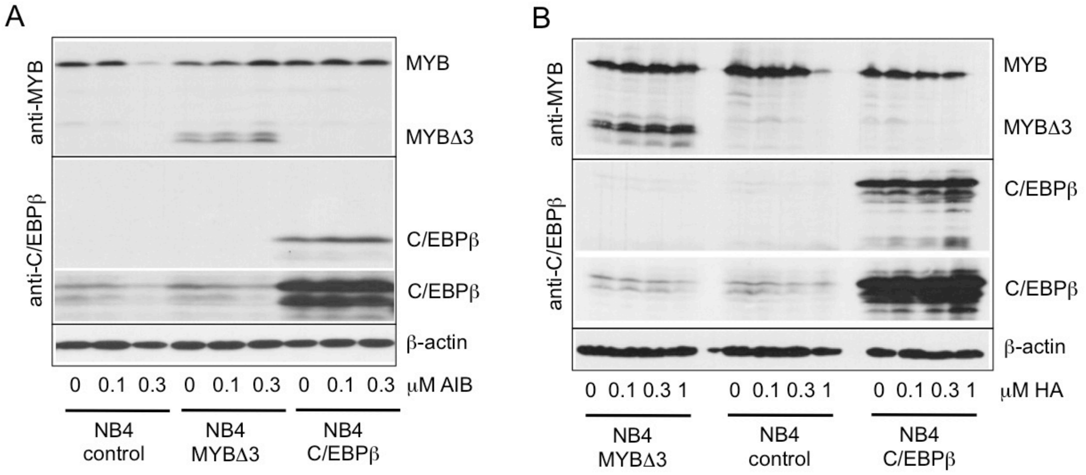

Supplementary Fig.8. **Ectopic expression of C/EBPβ or a C-terminally truncated version of MYB in NB4 cells.** NB4 cells infected with lentiviruses encoding human C/EBPβ (NB4-C/EBPβ), a truncated version of MYB (NB4-MYBΔ3) or an empty control vector (NB4-control). Cells were treated different concentrations of AIB (A) or HA (B) for 3 days. Cell extracts were then analyzed by western blotting for expression of MYB, MYBΔ3, C(EBPβ and β-actin.

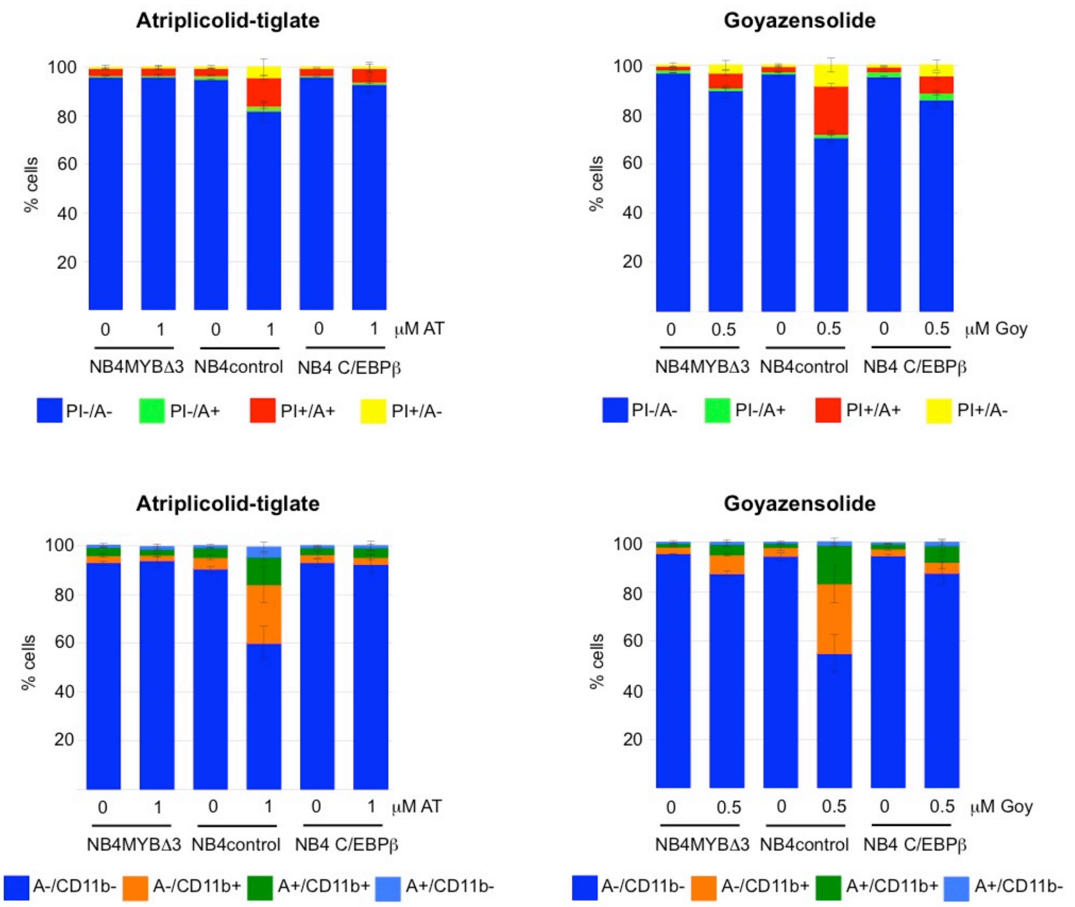

Supplementary Fig.9. **Ectopic expression of C/EBPβ or a C-terminally truncated version of MYB in NB4 cells counteracts AT- and GOY-induced differentiation and apoptosis.** NB4 cells infected with a control lentivirus (NB4control), a MYBΔ3-encoding lentivirus (NB4MYBΔ3) or a C/EBPβ-encoding lentivirus (NB4C/EBPβ) were treated for two days with AT or GOY at the indicated concentrations. The cells were then stained with annexin V and propidium iodide (upper panels), or antibodies against CD11b and annexin V (lower panels) and analyzed by flow cytometry. Bar charts in upper panels indicate the percentages (with standard deviations) of living (A-/PI-), early (A+/PI-) and late (A+/PI+) apoptotic and necrotic (A-/PI+) cells. Bars in lower panels indicate the percentage of CD11b positive and apoptotic cells (with standard deviations).

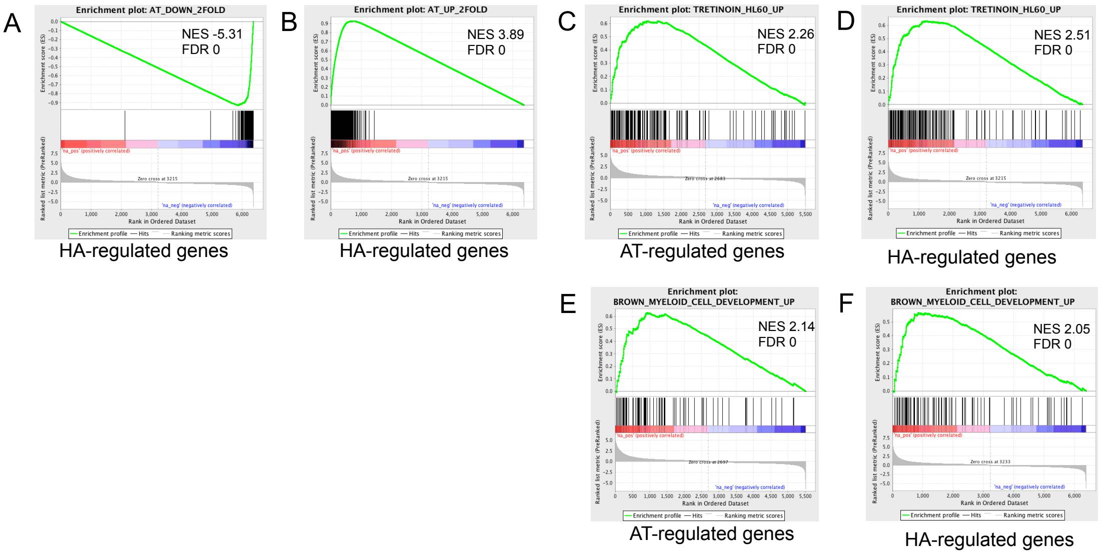

Supplementary Fig.10. **GSEA of the AT- and HA-gene expression signatures. A,B.** Genes down-regulated (A) or Up-regulated (B) two-fold by AT were plotted against HA-regulated genes. **C,D.** Genes up-regulated by tretinoin in HL60 cells were plotted against the AT- (C) and HA-regulated (D) genes. **E,F.** Genes up-regulated during myeloid development were plotted against the AT- (E) and HA-regulated (F) genes.

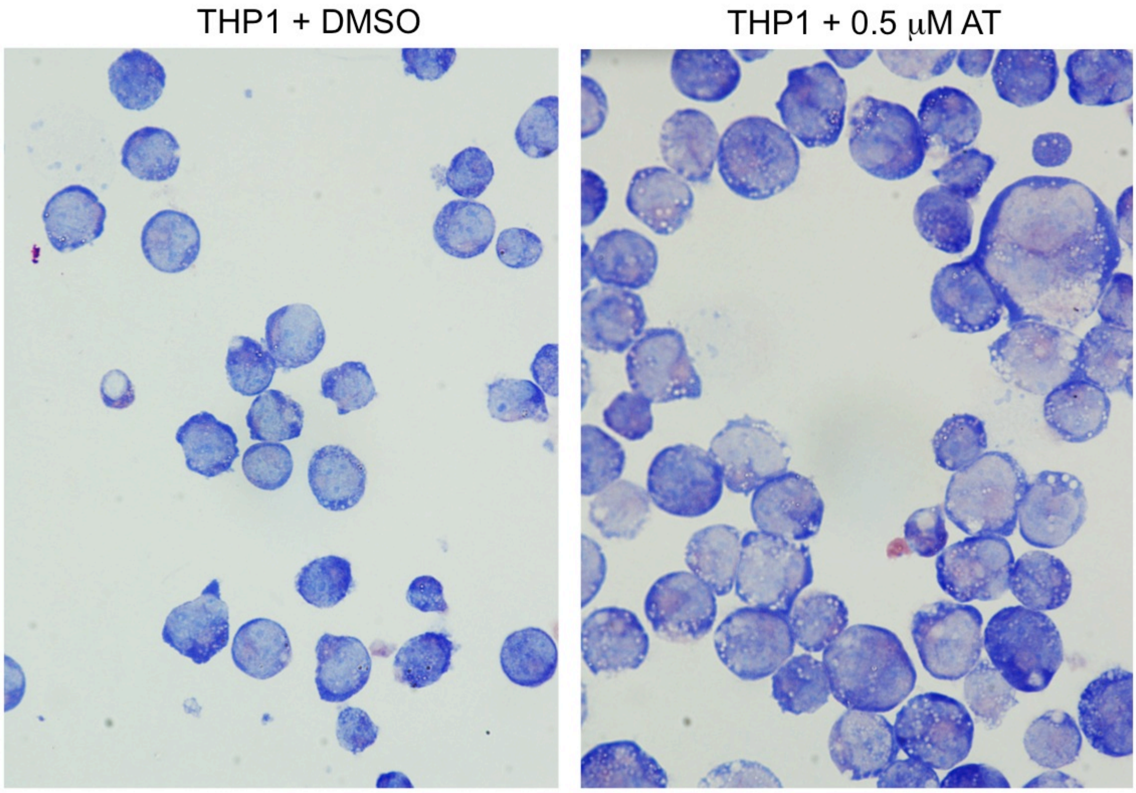

Supplementary Fig.11. **Morphology changes of THP1 cells induced by AT treatment.** THP1 cells treated with DMSO or 0.5 μM AT for 6 days. Cells were stained with May-Grunwald Giemsa.

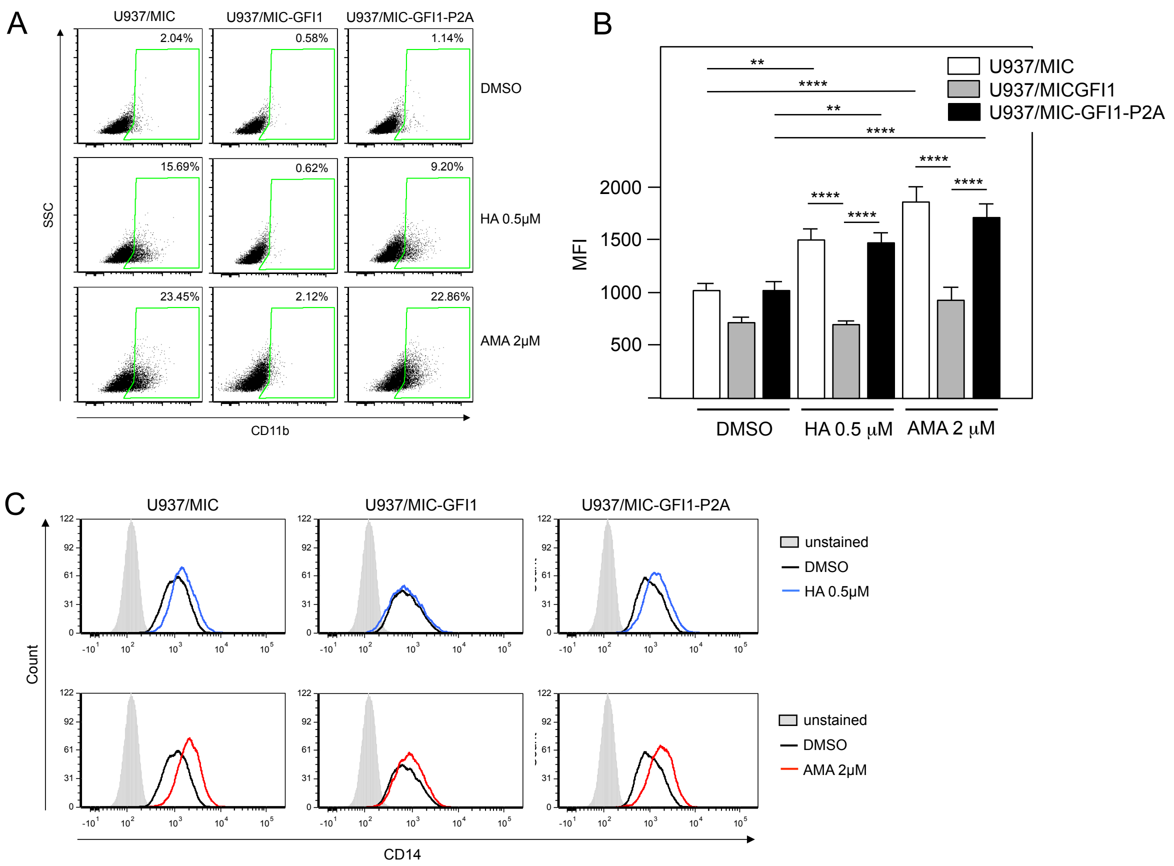

Supplementary Fig.12. **Effect of AMA and HA on CD11b and CD14 expression In U937/MIC, U937/MIC-GFI1 and U937/MIC-GFI1-P2A cells. A.** Analysis of CD11b expression of a representative flow cytometry experiment. **B.** Analysis of the mean fluorescence intensity (MFI) of cells stained with antibodies against CD14. Statistical significance was determined with one-way ANOVA with Tukey correction (**p<0.01, ***p<0.001, ****p<0.0001). **C.** Flow cytometry data of a representative experiment showing staining with antibodies against CD14 from U937 cell lines treated with HA (top) or AMA (bottom).

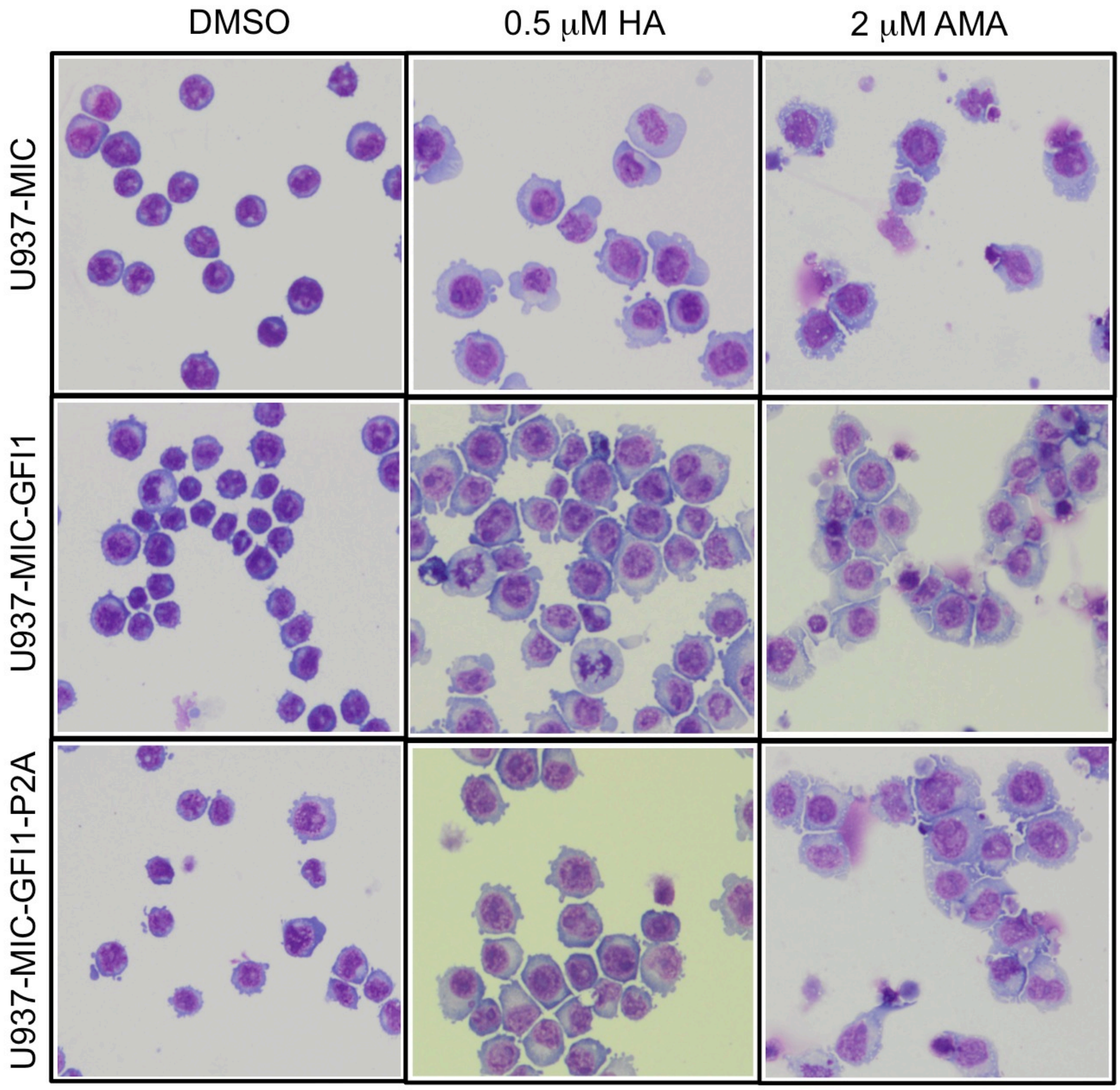

Supplementary Fig.13. **Effect of AMA and HA on the morphology of U937/MIC, U937/MIC-GFI1 and U937/MIC-GFI1-P2A cells.** Cells were treated for 96 h with DMSO, 0.5 μM HA or 2 μM AMA. Cells were stained with May-Grunwald Giemsa.

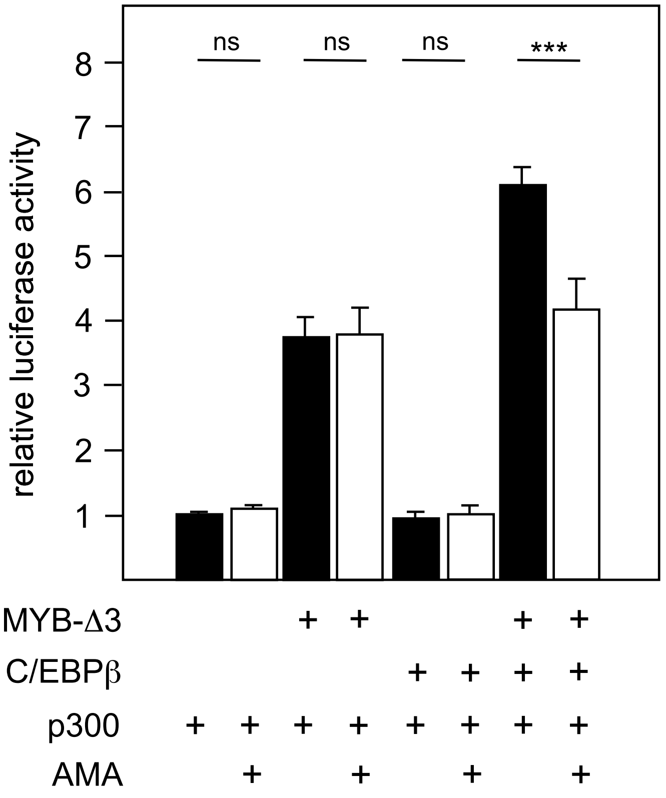

Supplementary Fig.14. **Effect of AMA on the *GFI1* promoter activity.** Reporter assays of the human *GFI1* promoter co-transfected with the indicated expression vectors in the absence or presence of 2 μM AMA. Asterisks statistical significance (***p<0.001, Student's t-test; ns: non-specific).

Supplementary table 1

Gene	Forward primer	Reverse primer
ABCC3	CAGAGAAGGTGCAGGTGACA	CTAAAGCAGCATAGACGCCC
ACTB	CGTCCACCGCAAATGCTT	GTTTTCTGCGCAAGTTAGGT
CCL2	CCCCAACATCACTCTCCACC	ATGGTCTTGAAGCTGTGGGG
CD11b	CCTGGTGTTCTTGGTGCCC	TCCTTGGTGTGGCACGTACTC
CD11c	CCGATTGTTCCATGCCTCAT	AACCCCAATTGCATAGCGG
CD14	AAAGGACTGCCAGCCAAGCT	GATTCCCGTCCAGTGTCAGGT
CLSTN2	CAAAGAGCCAGCCTACAAGG	CAAAAGGCACATCTGTGGTG
cMYC	GCCGATCAGCTGGAGATGA	GTCGTCAGGATCGCAGATGAAG
CYP4F3	GGGCTTTAAGGTCTGGATGG	GGGCTTCAGGATGTTGAAAT
ETV1	GTCAGCGTGGGAGAAATTGT	ACTGCAGGCAGAGCTGATTT
GFI1	GCTCGGAGTTTGAGGACTTC	ATGGGCACATTGACTTCTCC
GPR34	TGTCTAGGGAAGCTTGGGGT	GTCGCAACCTTTCACCGTTT
HDAC9	GAGCATATCAAGGGGCAGTG	CTTCAAGTTGGGCTCAGAGG
IGSF23	GGGCTTTAAGGTCTGGATGG	GGGCTTCAGGATGTTGAAAT
LRIG1	CACTTGCGCTGGGGACTC	CTCGATCATCAAATGTAGGCTCC
KIT	TGATTTTCCTGGATGGATGG	TGGGATTTTCTCTGCGTTCT
MAFB	ACGTGAAGAAGGAGCCACTG	TGTGTCTTCTGTTCGGTCGG
MCM7	TGAAGGACTACGCGCTAGAG	CCTCAAACGGCCAATCCC
MS4A3	GTGGACTTGGGAGGAAAGCC	GGACACCAGGGCAGAGTAAC
PRTN3	AGAGGAGCTTGACCGTGG	GATCTGCAACCCGGTCAGT
RTKN2	GACAGTTCGCGTTGGAGATG	TCCATATTCCTTCTCGCATTCG
SERPINI2	AATGGAGTTGGCCTTGATCG	TGCTGAACTAAACAACCTGATGA
SPP1	GCCGAGGTGATAGTGTGGTT	TGAGGTGATGTCCTCGTCTG

Supplementary table 1. Forward and reverse real-time PCR primer sequences.
